# Optimization of Medication Delivery Drone with IoT-Guidance Landing System Based on Direction and Intensity of Light

**DOI:** 10.3390/s22114272

**Published:** 2022-06-03

**Authors:** Mohamed Osman Baloola, Fatimah Ibrahim, Mas S. Mohktar

**Affiliations:** 1 Department of Biomedical Engineering, Faculty of Engineering, Universiti Malaya, Kuala Lumpur 50603, Malaysia; 17222412@siswa.um.edu.my (M.O.B.); mas_dayana@um.edu.my (M.S.M.); 2 Centre for Innovation in Medical Engineering (CIME), Faculty of Engineering, Universiti Malaya, Kuala Lumpur 50603, Malaysia; 3 College of Engineering and IT, Ajman University, Ajman P.O. Box 346, United Arab Emirates; 4 Centre for Printable Electronics, Universiti Malaya, Kuala Lumpur 50603, Malaysia

**Keywords:** IoT, guidance landing system, light direction, light intensity, drone, face recognition

## Abstract

This paper presents an optimization of the medication delivery drone with the Internet of Things (IoT)-Guidance Landing System based on direction and intensity of light. The IoT-GLS was incorporated into the system to assist the drone’s operator or autonomous system to select the best landing angles for landing. The landing selection was based on the direction and intensity of the light. The medication delivery drone system was developed using an Arduino Uno microcontroller board, ESP32 DevKitC V4 board, multiple sensors, and IoT mobile apps to optimize face detection. This system can detect and compare real-time light intensity from all directions. The results showed that the IoT-GLS has improved the distance of detection by 192% in a dark environment and exhibited an improvement in face detection distance up to 147 cm in a room with low light intensity. Furthermore, a significant correlation was found between face recognition’s detection distance, light source direction, light intensity, and light color (*p* < 0.05). The findings of an optimal efficiency of facial recognition for medication delivery was achieved due to the ability of the IoT-GLS to select the best angle of landing based on the light direction and intensity.

## 1. Introduction

Unmanned air vehicles (UAV), which are commonly known as drones, are classified globally based on their weight, flight range, flight speed, and payload. Drone applications are becoming an increasingly important part of many services. Drones are playing a significant role with the advances in technologies in the Fourth Industrial Revolution (4IR). The investment in the drone industry reached 127 billion USD and is expected to offer a hundred thousand job opportunities in 2025 [1].

Drone applications in the medical field to enhance healthcare services to patients have grown rapidly. Recently, the spread of COVID-19 has resulted in the application of drones in the medical field, such as street disinfection to stop the spread of the virus. In addition, a thermal camera has also been used to detect the abnormal temperature of an infected person in a crowded area and lead to action to isolate him [2].

Homecare, telemedicine, and lockdowns have encouraged medicine delivery to patients using drones. Social distancing is one of the most important factors in stopping the spread of the virus, and drones are playing a significant role because their cameras can monitor any violations; loudspeakers attached to a drone can also be used to encourage people to keep a safe distance [2].

Research from Beck has also focused on drones delivering anti-allergy injections that help in saving lives. The study focuses on the effect of the injection ingredients in different scenarios, such as frequencies of vibration, environmental temperatures, and flight time [3].

Balasingam reported that NASA had tested the first government-approved medication delivery drone to deliver medication for asthma, hypertension, and diabetes [4].

Drones are improving Emergency Response Time (EMS). Researchers have proposed a network of 500 drones to serve cardiac patients with an automated external defibrillator (AED) faster, and the results indicated that the drones could improve the response time by 5 min, which reduces the response time from 7 min and 42 s to 2 min and 42 s [5]. Simulation studies using drones and ambulances were conducted in Canada to measure the difference in response times in the countryside. Six simulations were conducted where the distance between the base and the victim was between 6 and 20 km. In all the simulations, the drone arrived at the victim earlier than the ambulance. In the slower response, the drone arrived earlier than the ambulance by 1 min and 48 s, and the faster response of the drone reached the victim earlier than the ambulance by 8 min [6]. Another study concluded that 100 drones could improve the response time and reduce it by up to 6 min in the city and 10 min in the countryside [7]. A literature review done by researchers showed a significant difference between distance and time for delivering a blood sample between hospitals and laboratories in three cities in Switzerland. All four tests prove that drones are faster than a traditional delivery method regardless of the distance between hospitals and laboratories. In the first test, in Lugano, the distance between the hospital and laboratory was 3.6 km by car and 1 km by drone, the drone delivery time was 3 min, while by car it was more than 30 min. In the second test in Zurich, the distance by car and drone were almost the same, the distance between the hospital and laboratory was 6 km by car and 5.8 km by drone, the drone delivery time was 7 min while by car it was more than 20 min. The third test was done in the same city where the distance by drone was almost double the distance by car, the drone delivery time was 7 min while by car was 10 min [8].

The cameras on drones are used for surveillance, facial recognition, object detection, photography, photogrammetry, etc. [9,10]. Facial recognition technology refers to a branch of computer vision used in many applications, such as surveillance, access control, attendance, access privileges, etc. [9,11]. A recent review showed that the first computer-based research conducted in this field was in 1964 and focused on 20 face parameters, including the dimension of eyes and mouth, followed by decades of technological enhancement where most of the leading companies, such as Facebook and Amazon, developed facial recognition applications for their services [12]. The COVID-19 pandemic has forced everyone to wear masks. A research group from Wuhan, China, proposed two types of facial recognition. The first type focuses on detecting the proper wearing of a mask, while the second focuses on facial recognition with the mask [13]. This technology is trending because of the rules and relegations that enforce the wearing of masks, which affects traditional facial recognition. The accuracy of the facial recognition during the drone’s hovering is based on the angle of depression as well as the distance between the drone and the face [14], while for 2D facial recognition, the accuracy is affected by face pose, light intensity, age, physical appearance, and cosmetics [12]. The most advanced 3D facial recognition is better because it is less sensitive to pose and lighting variation. However, an active and passive technology is required to scan a 3D object by specific cameras such as Microsoft Kinect and Bumblebee XB3 [15].

Currently, facial recognition by drones is used in surveillance. Even though the current facial recognition technologies used on the drone are able to recognize faces, the accuracy of the facial recognition during a drone’s hovering is based on the angle of depression as well as the distance between the drone and face [14] and the face poses, light intensity (inclusive of low light, improper illumination, and weather), age, physical appearances, and cosmetics [12,16]. Researchers reviewed the five most common facial recognition algorithms. First, the Principal Component Analysis (PCA) is the easiest to use but it is very sensitive to light and shadow, while the Linear Discriminant Analysis (LDA) is most efficient for illumination issues. The Local Binary Pattern Histogram (LBPH) is the best for a challenging environment, but it is slower because of the processing time as well as the efficiency of LBPH dropping during extreme brightness. The Elastic Bunch Graph Matching (EBGM) is the best algorithm for the posing challenge, but at the same time, it is sensitive to light. Finally, the Neural Networks is the most accurate algorithm for facial recognition, but it requires more data compared to most algorithms. This paper states that three out of five algorithms are sensitive to light. The process of the facial recognition system is divided into three parts. The first step is to start with face detection by using many techniques such as the Viola–Jones detector and Principal Component Analysis (PCA) [17]. Second is the extraction of the face’s features such as face’s shape, mouth, nose, and eyes by extraction tools such as Eigenface and Local Binary Pattern (LBP). Third, the extracted face’s features are compared with faces in the database [17].

The Internet of Things (IoT) was invented in 1999 by Kevin Ashton. He added RFID to all lipsticks to communicate together. Today, companies and communication service providers are competing to offer IoT solutions to individuals and corporations. Students, researchers, and entrepreneurs are exploring the challenges and opportunities to develop new innovative products and solutions. Researchers have reviewed IoT applications for smart home, industry, healthcare, agriculture, environment, and transportation [18]. IoT mobile apps are believed to bring assistance and are able to facilitate access to information and organize processes and activities [19]. Developing research in recent years has been the pursuit of designing smart drones with the addition of IoT sensors. Drones embedded with IoT may be made more beneficial and effective by using an assembly of technologies such as sensors, transmitters, and cameras for an array of various and advanced applications [20].

The availability of easy-to-use IoT modules, a variety of online platforms for IoT, and availability of Internet connectivity indoors and outdoors increase the demand for IoT technology globally. Technology improves the battery’s lifetime to more than 10 years like the Narrow Band IoT (NB-IoT) and eMTC [21].

IoT applications on drones are used for remote sensing and sending the data by Internet. The 5th generation of mobile technology known as 5G also has many advance features that support IoT applications. 5G provides a better data transfer rate up to 100 times compared to previous network generations and download speed up to 20 Gbps plus a very low latency such as 1 ms latency which is useful for autonomous drones [22,23].

Monitoring the vital signs of the human body in the healthcare sector is required to save a life, better diagnose, and provide better treatment to the patient. IoT helps healthcare providers to enhance the quality of their services which reflects in a healthy community and reduces the risk of medical error. On the other hand, we should not neglect the privacy of the patient’s data by keeping critical data in an online database [24].

The basic structure of IoT system is to connect the IoT module with a sensor to send data over the Internet. The collected data by drone’s sensors are processed either on the board or sent by the Internet to the base station. The drone’s IoT sensors are divided into flight control, data collection, and communication sensors [25]. Medicine Box is developed by a group of students who connected by IoT to the Internet. This Intelligent Box sends a notification to the mobile application in the patient mobile phone to take his medicine on time [26].

Today, researchers are monitoring data from sensors on the mobile phone application. Groups of researchers are monitoring and storing the pressure on the heel from sensors in shoes. The pressure values are sent by PIC Microcontroller via Bluetooth [27]. A recent study at Taif University in Saudi Arabia monitored the acquired data from weather sensors on the Blynk app. The BME280 weather station sensors are mounted on a drone to collect pressure, temperature, altitude, and humidity as well as a transmitter to send a video from a thermal camera. Blynk is IoT mobile app that works on mobile phones and tablets for Android and iOS [28].

The standard Huskylens facial recognition camera has a Kendryte K210 processor with a 2 megapixel camera; the company stated that their system uses the You Look Only Once (YOLO) algorithm which works between one to two frames per seconds. The Huskylens camera is thirty time faster than YOLO but the company did not share any details about it [29,30]. Kendryte K210 is a low-power consumption system on chip (SoC) and powerful for facial recognition and detection as well as image classification and object detection. It uses the Convolutional Neural Network (CNN) for machine vision. It performs a real-time facial recognition up to 60 fps [31]. Redmon et al. introduced his YOLO for object detection in 2016. YOLO is widely used for object detection. He stated that the fast YOLO can work up to 155 fps while the regular YOLO works up to 45 fps [32].

Thus, the objective of this paper is to optimize the facial recognition ability of a developed medical delivery drone based on the direction and intensity of light. The main challenge for the drone’s facial recognition occurs when the sun has a sharp angle during the sunrise in the early morning and from afternoon to sunset, as well as during the night when there is insufficient light, especially when the light intensity is between 20 and 50 Lux. The light intensity and direction of the light affect the accuracy of our facial recognition. The enhancement includes the development of a Guidance Landing System (GLS) incorporate with IoT mobile apps.

### Background

From the literature review, most of the facial recognition algorithms are developing their software to overcome illumination issues. Our contribution in this paper is to develop a hardware system along with an IoT mobile app to enhance the efficiency of medicine delivery.

**Figure 1:**
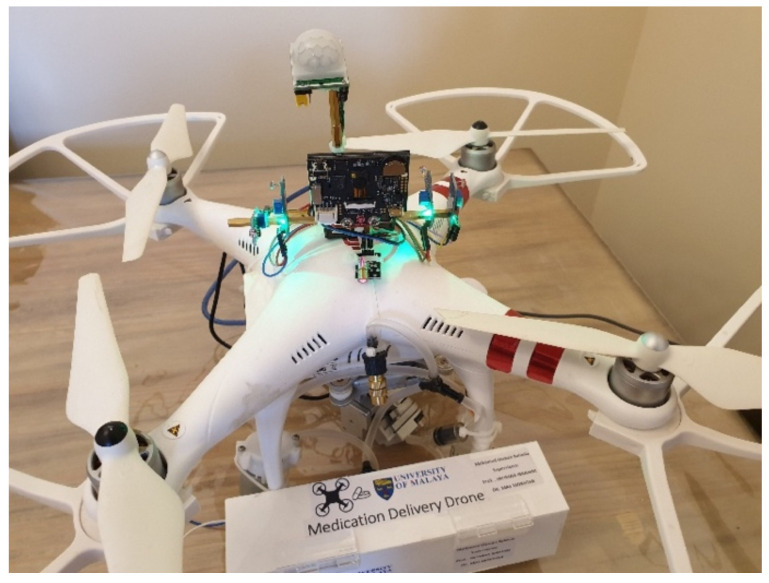
Drone with facial recognition and medical delivery box.

**Figure 2:**
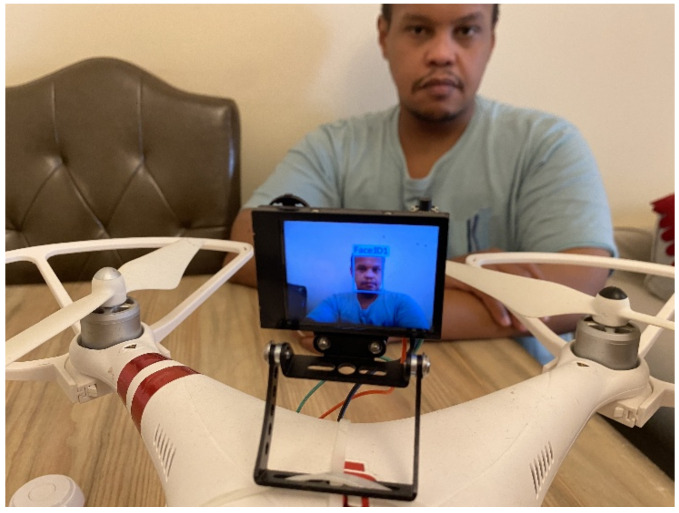
Facial recognition to identify specific user on the medical delivery drone.

A medical delivery drone with a facial recognition ability has been developed as shown in Figure 1. The medical delivery drone is equipped with a secure mechanical delivery box (DB) inclusive of a sanitizing unit (SU) that sprays sanitizer to avoid the risk of the spread of the COVID-19 virus during the delivery. The developed drone can deliver medicine in a very secure way by using facial recognition to identify the user (Figure 2). The drone was designed to be interactive with the user and uses voice commands to communicate with the user. The drone is equipped with a Huskylens facial recognition artificial intelligence (AI) camera that is used for the facial recognition process to identify the patients and to proceed with the delivery process according to the user profile.

The accuracy of the delivery is based on the accuracy of the facial recognition, which is affected by the distance of face detection (the distance between the face and camera). A positive relationship exists between the light intensity and detection distance, the detection distance increases when the light intensity increases and vice versa. To solve this issue, this paper presented the design of the GLS to help the operator or autonomous system to select the best landing area. Five light sensors were assembled on GLS to detect the light direction and intensity and evaluate the system’s accuracy.

**Figure 3:**
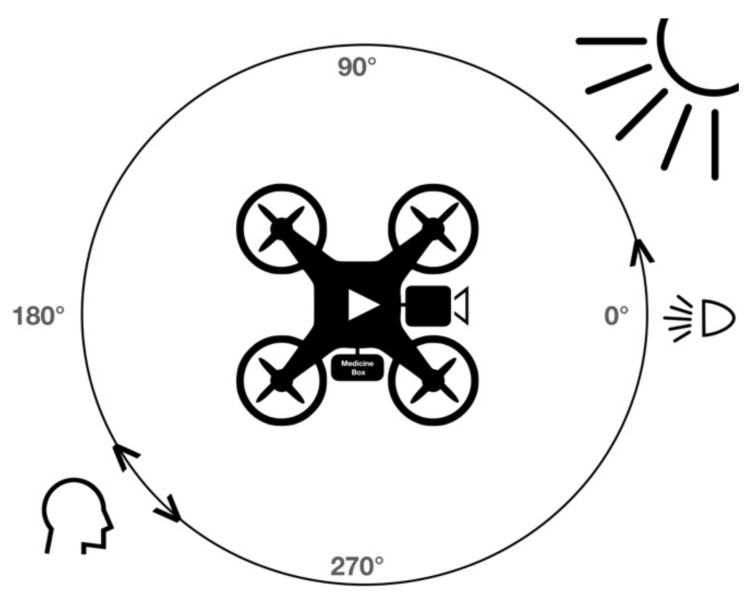
Drone rotates (yaw) 360° around vertical axis at the same location.

A multi-rotor drone has the ability to land at the same location with freedom of rotates (yaw) 360° around the vertical axis as shown in Figure 3. Outdoor facial recognition has extreme challenges based on the variation of environmental conditions. A set of experiments were conducted to improve the quality of facial recognition and detection.

This paper presents a precision medication delivery system using drones and IoT GLS as shown in Figure 2. The drone uses facial recognition to identify the user as shown in Figure 1. In this paper, Section 1 covers a literature review about drones, IoT, and facial recognition. Then, Section 2 focuses on the hardware and software of the developed drone’s system. Next, Section 3 presents the result. After that, Section 4 is for the discussion. Finally, Section 5 concludes the findings of the study.

## 2. Materials and Methods

### 2.1. Development of Facial Recognition System

**Figure 4:**
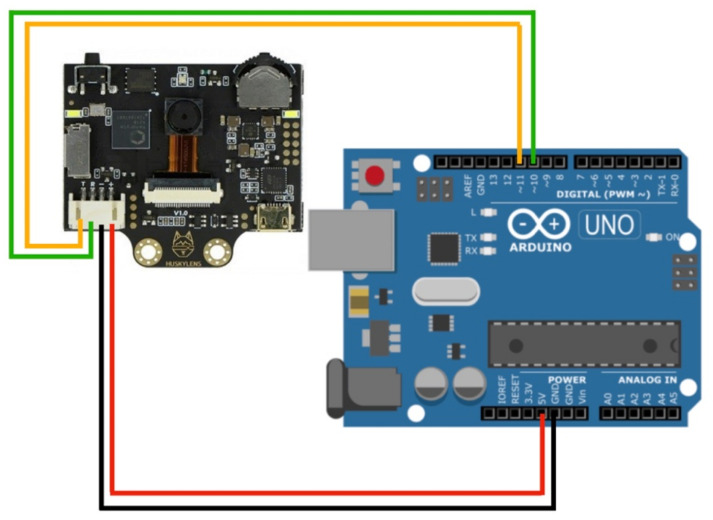
Circuit connections diagram for the Huskylens facial recognition camera.

The Huskylens facial recognition camera (DFRobot Electronics, Shanghai, China) has a Kendryte K210 processor. It is a very light camera that has a facial recognition function and does not require any Internet connection. It is compatible with the Arduino development board (Arduino S.R.L., Via Andrea Appiani, 25, 20900 Monza MB, Italy) via Universal Asynchronous Receiver Transmitter (UART) or Inter-Integrated Circuit (I2C). The first development board reads from the Huskylens facial recognition via UART serial communication at a 9600 baud rate as shown in Figure 4. The development board reads the user ID from the camera and matches it with a pre-defined user’s identity. It operates by 5 volts. Digital pins 10 and 11 are connected to Rx and Tx of the Huskylens camera, respectively. Huskylens has a Multiple Faces Learning function. Thirty photos were obtained from our volunteers. Faces were given ID numbers 1 to 3. ID = 0 is given for any undefined faces. ID = 0 code is helping us in the detection of any face presence. Accordingly, a specific response was programmed in Arduino Uno to the user ID. The delivery box (DB) will open for the defined ID 1, 2, and 3 to deliver the medicine, while the Voice Guiding System (VCGU) will play a greeting voice message by his/her name for ID 1, 2, and 3 or will play a voice message for ID 0 to move away from the drone.

### 2.2. Development of Guidance Landing System (GLS)

**Figure 5:**
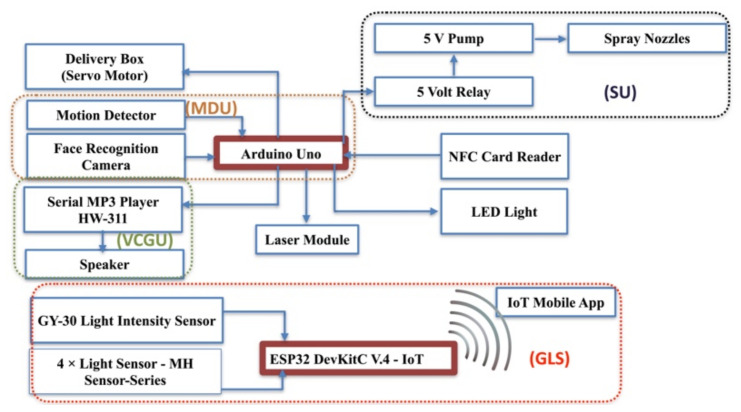
Medical delivery drone enhanced components for Guidance Landing System.

To improve the medical delivery drone with GLS, the system was enhanced with the ESP32 DevKitC V4 board (Espressif Systems Co., Ltd., Shanghai, China). This microcontroller board has a dual-core processor, 12-bit ADC pins, and Wi-Fi which is useful for IoT applications. In addition, four MH Light-Dependent Resistor (LDR) sensors are connected to 12-bit ADC pins (Figure 5).

**Figure 6:**
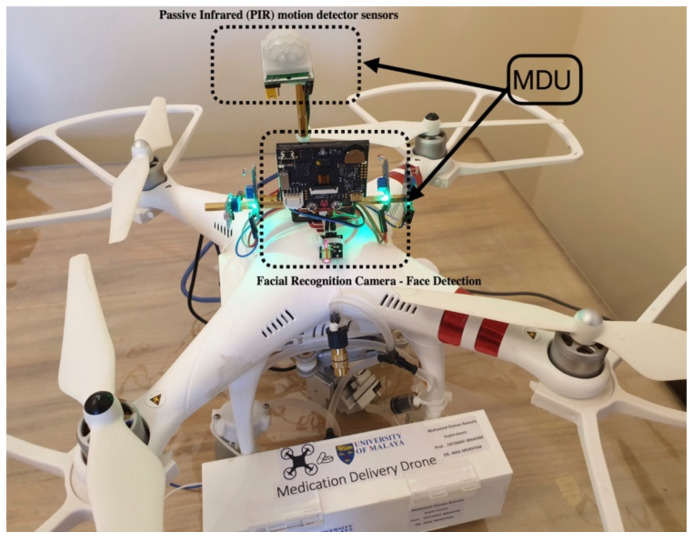
Guidance Landing System (GLS) and Motion Detection Unit (MDU).

A Motion Detection Unit (MDU) was also implemented with the GLS to save the power of the drone and ensure the safety of users (Figure 6). The medical delivery drone is set to be in standby mode when no motion is detected beside the drone and disable the process of the delivery box when there is human movement beside the drone, and back to standby mode when the person moves away from the drone. The take-off mode will start when there is no presence of a human close to the drone. The MDU combines a Huskylens camera and Passive Infrared (PIR) motion detector sensors.

The GLS is equipped with a Global Positioning System (GPS) to guide the drone to the outdoor landing position close to the patient’s home. The patient receives a notification on the estimated delivery time during the process of scheduling a delivery. Before the delivery, SMS and emails notification will be sent to the patient’s registered mobile phone number and email. Delivery is based on the patient’s identity. In the case of an elderly, sick, or busy patient, the database of faces must be updated with the facial recognition details for two of the patient’s relatives. When the drone reaches the patient’s location, the SMS and email will be sent to the patient’s registered mobile phone number and email, along with a siren alarm.

The drone is equipped with a Voice Guiding System (VCGU) to be an interactive system with the user, as shown in Figure 5. A Serial MP3 player model HW-311 is used to give a voice command to the user. Serial communication is used to send an array of 8 bytes to play a specific audio file. Each array contains starting and ending bytes, version, array size, command, enable and disable feedback, and two bytes of data. For instance, the first audio message is for greeting the user, and the second message will request that the patient stand in front of the camera.

### 2.3. Development of IoT Mobile App for Guidance Landing System (GLS)

**Figure 7:**
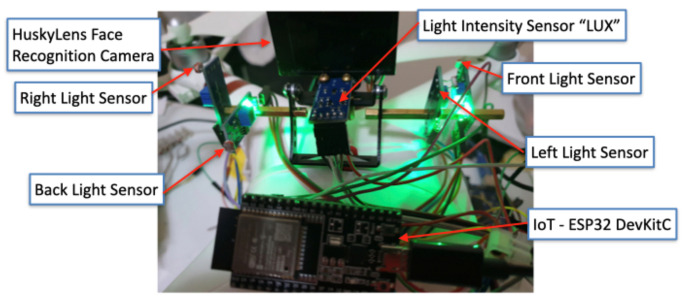
Guidance Landing System (GLS) hardware components.

LDR sensors are used to measure the light intensity from four directions as shown in Figure 7. A light intensity sensor gives a numerical value of the light intensity in Lux. The sensor is aimed opposite to the facial recognition camera to measure the light intensity that falls on the subject’s face.

**Figure 8:**
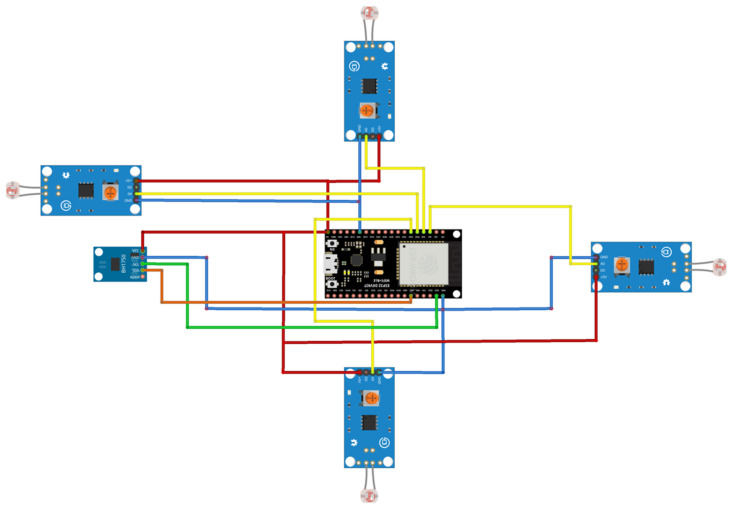
Part of Guidance Landing System (GLS) circuit connections diagram.

**Figure 9:**
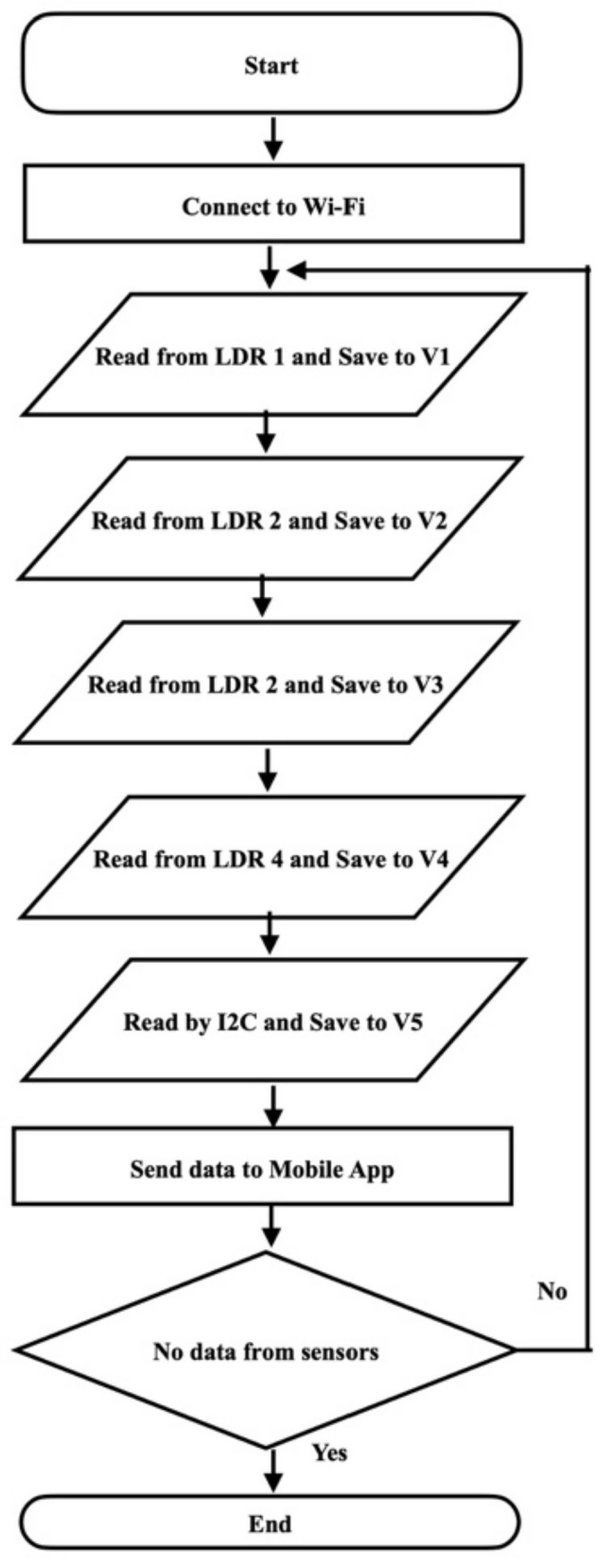
Flowchart of Guidance Landing System. Notes: LDR—Light-Dependent Resistor; V1, V2, V3, V4, and V5 are the virtual pins in the IoT mobile apps.

The IoT development board reads from the sensors and sends the data to IoT mobile applications as shown in Figure 8. The IoT development board connects to the Wi-Fi network. The development board reads from five sensors as shown in the flowchart in Figure 9. The first light intensity sensor sends light intensity in lux to the development board. Four photoresistors compare the light percentage from four directions. The light percentage Equation (1) from four directions is then sent to a mobile application to compare the light intensity from all angles.

(1) $$\%\;of\;the\;light=\frac{Analog-to-Digital\;Converter\;\left(ADC\right)\;Value\times 100}{2^{12}-1}$$

A Blynk mobile app was developed to show the real-time light intensity and compare the light intensity from all directions in terms of percentages. The values of the five sensors are saved on five virtual pins after the calculations. In the mobile apps, the five virtual pins are selected to display the values of the five sensors.

### 2.4. Experimental Methods to Study the Effect of Light Direction Using Uncontrolled Light Source

Multiple scenarios were simulated to determine the relationship and effect of light direction on facial recognition quality using an uncontrolled light source. Lux (lx) indicates the amount of light intensity in Lumen per square meter.

The distance between the LED light source and drone was fixed at 111 cm, and the LED light source intensity was fixed at 580 Lux.

**Figure 10:**
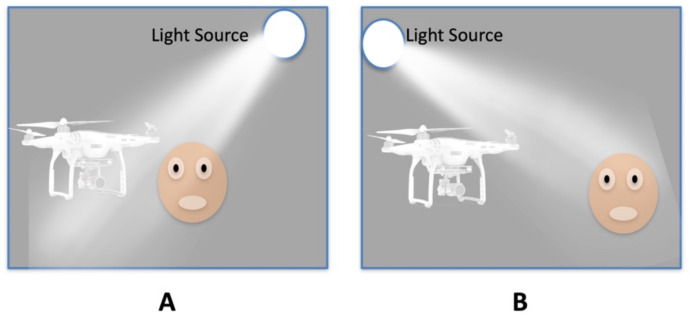
Guidance Landing System (GLS) and direction of light, (**A**) the light is falling on the back of the face; (**B**) the light fell on the face.

*Scenario A*:

In a dark room with a single LED light source where the light falls on the back of the face, as shown in Figure 10A.

*Scenario B:*

In a room with a light intensity of 27.5 Lux with a single LED light source where the light is falling on the back of the face, as shown in Figure 10A.

*Scenario C:*

A dark room with a single LED light source when the light is falling on the face, as shown in Figure 10B.

*Scenario D:*

A room with a light intensity of 27.5 Lux with a single LED light source when the light is falling on the face, as shown in Figure 10B. The detection distance of the subject’s face using the drone camera was observed for all four scenarios.

### 2.5. Experimental Methods to Study the Effect of Light Intensity and Color Using Controlled Light Source

**Figure 11:**
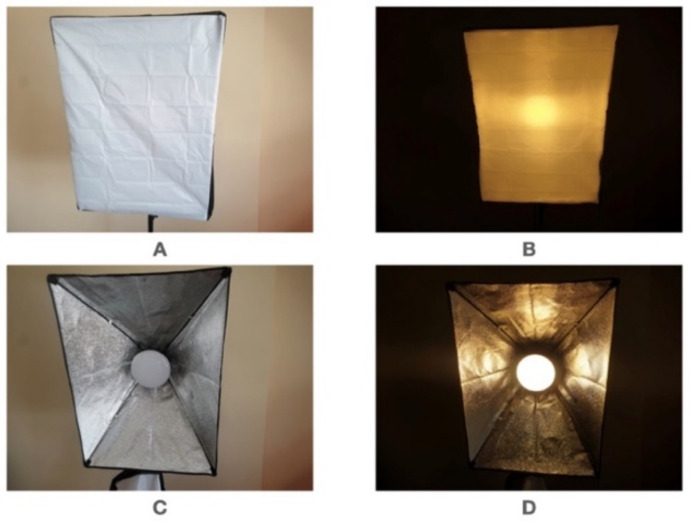
(**A**) Light source with diffuser when light off. (**B**) Light source with diffuser when light on. (**C**) Light source without diffuser when light off. (**D**) Light source without diffuser when light on.

In this experiment, a controlled light source was used to control light intensity and light color. The controlled values were 3200 K and 5500 K. Two types of light sources were used (Figure 11), a light source with a diffuser and a light source without a diffuser. The Kelvin (K) is a light color temperature. Lower light temperature indicates reddish color while the higher light temperature is bluish.

**Figure 12:**
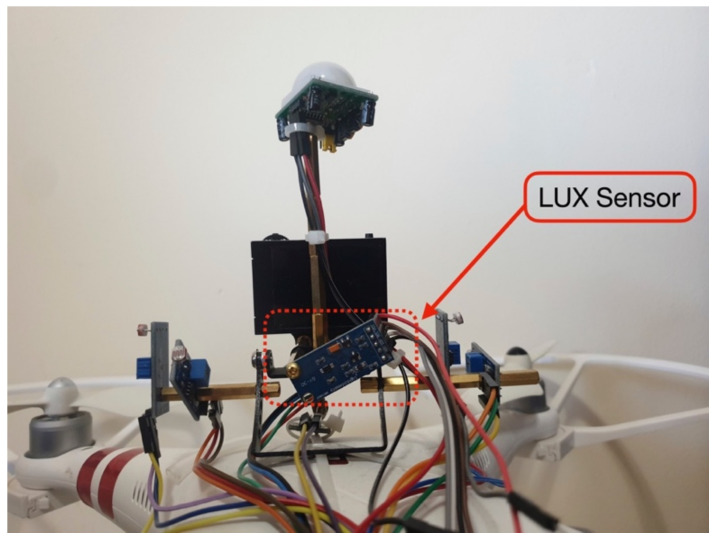
The sensor (Lux) was moved to the backside of the drone to detect the light from the controlled source.

The controlled light direction was aimed at the backside of the drone’s camera; thus, the sensor (Lux) was moved to the backside of the drone, as shown in Figure 12.

The first group of tests was conducted using a controlled light source with a diffuser, as shown in Figure 11A,B. The light intensity and distance were measured when the subject’s face was recognized at five levels. The five levels of tests were repeated for 3200 K and 5500 K.

The tests were repeated for the second group of tests using a controlled light source without a diffuser, as shown in Figure 11C,D, and again the light intensity and distance were measured when the subject’s face was recognized at five levels.

The following Pearson correlation coefficient was used to find the correlation between the distance of detection and intensity of the light in different scenarios.

(2) $$r=\frac{\sum_{i=0}^{n}\left(x_{i}-\bar{x}\right)\left(\left(y_{i}-\bar{y}\right)\right)}{\sqrt{\sum_{i=0}^{n}{\left(x_{i}-\bar{x}\right)}^{2}}\sqrt{\sum_{i=0}^{n}{\left(y_{i}-\bar{y}\right)}^{2}}}$$

## 3. Results

By enhancing the IoT-GLS system, the medical delivery drone’s operator or autonomous system was able to select the best angle of landing based on the light direction and intensity to ensure the optimal efficiency of facial recognition. The drone was able to land and switch off all rotors in the patient’s location based on the GPS coordinates. Then, the drone was able to detect any person near the drone’s landing area through the motion detector and facial recognition camera and can request the patient to stand in front of the camera to verify the patient’s identity. For an easier user experience, a controllable laser light was used to point a red laser light to the ground in front of the camera and guide the patient to the right location in front of the camera, which is close to the delivery box. Then, according to the patient’s identity, the drone was able to greet the patient by his/her name using a voice. Then, the delivery box was opened for the patient to collect his/her medicine. Finally, the box closed, and the sanitizing process started to ensure that the spread of any viruses and bacteria to the patients and the operators can be eliminated. In case of detecting of any unauthorized person in front of the camera, the drone starts with a warning message that requests the person to move away. In case the violator does not respond within 10 s, the alarm sounds and a photo is sent to the operation center.

The accuracy of the Huskylens facial recognition camera was tested on three volunteers as well as 5000 faces from the Flickr-Faces-HQ dataset (Q) were used to check the accuracy of the system. The majority of the faces were undefined to calculate the accuracy of face detection. The photos of the three volunteers, representing 0.596% of the total number of 5030 photos, were identified.

The Huskylens camera has two features; the first feature is detecting the presence of a human face, while the second is identifying the identity of the face. The first test included 5000 photos from the FFHQ dataset for human face detection by the Huskylens camera. The system successfully detected the presence of 4997 faces with 99.94% accuracy. Only 3 faces out of 5000 were not detected. Two faces were not detected because the hair was covering a part of the faces, while the third face was covered with a hat. The false positive ratio for face detection is 0.06%.

In the second experiment, 30 face pictures of the three volunteers were used to check the accuracy of the system. The facial recognition camera detected 29 faces. The one undetected face had a low-quality picture. As a result, the percentage of error reached 3.33%.

The first two tests were conducted with the three volunteers’ photos plus 5000 photos from the FFHQ dataset. The overall percentage of our two tests’ accuracy reached 99.92% in a controlled environment with perfect lighting conditions.

**Figure 13:**
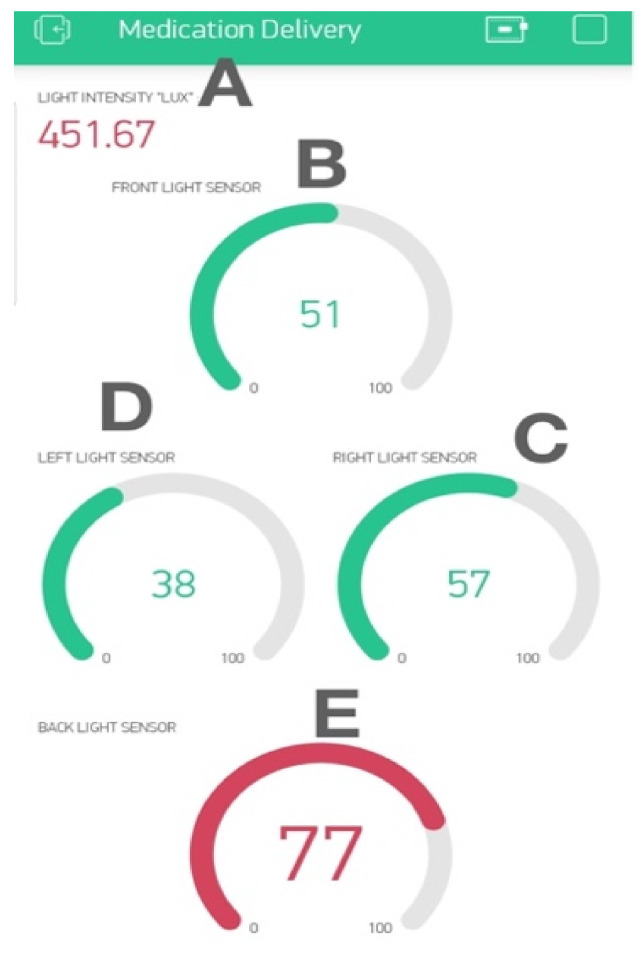
IoT mobile app of Guidance Landing System (GLS). (**A**) The upper numerical value shows the light intensity the backside. (**B**) The light intensity percentage of the front-side sensor. (**C**) Light intensity percentage of the right-side sensor. (**D**) Light intensity percentage of the left-side sensor. (**E**) Light intensity percentage of the back-side sensor.

The light percentage from four directions was then sent to the IoT mobile application to compare the light intensity from all angles, so the best angle for landing could be chosen. The developed IoT mobile app is shown in Figure 13. The upper numerical value shows the light intensity at the backside (Figure 13A), the light intensity percentage of the front-side sensor (Figure 13A), the light intensity percentage of the right-side sensor (Figure 13C), the light intensity percentage of the left-side sensor (Figure 13D), and the light intensity percentage of the back-side sensor (Figure 13E).

In the study of the effect of light direction using uncontrolled light source tests, in scenario A, where the facial recognition efficiency was tested in a dark room with a single LED light source where the light direction is falling on the back of the face, the camera did not detect the face even from a very close distance. In scenario C, where the facial recognition efficiency was tested in a dark room with a single LED light source, when the light direction fell on the face of the subject, the camera was able to detect the subject’s face from 192 cm.

In scenarios B and D, the facial recognition efficiency was tested in the room with the light at 27.5 Lux, and the LED light source was kept at the same position. When the light direction fell on the back of the subject’s head, the camera detected the face from 60 cm (scenario B). However, in scenario D, when the light direction fell on the subject’s face, the camera detected the subject’s face from 207 cm.

The results of the study of the effect of light direction using uncontrolled light source scenarios show that the IoT-GLS improved the distance of detection by 192% in a dark environment with a single light source when the IoT-GLS chose the best angle when the direction of light fell to the face of the subject. Furthermore, the test was repeated in a room with low light intensity (27.5 Lux) plus a single LED light source. The measurement exhibited an improvement in face detection distance up to 147 cm by using the IoT-GLS.

To improve the measurement of the light intensity that falls toward the face, the direction of the sensor changed from upward to the back of the drone (Figure 12). The second test was conducted to study the effect of light intensity and color by controlling the light source.

The first group of controlled light source tests was conducted using a controlled light source with a diffuser. The light intensity was measured at five levels. The five levels of tests were repeated for 3200 K and 5500 K. The distance when the subject’s face was recognized and measured at each test is shown in Table 1.

Table 2 shows the collected data for the light sources without diffuser tests. The highest light intensity was at 5500 K direct light, which reached to 500.83 Lux and the detection distance reached 184 cm.

Then, a set of four experiments was conducted to measure the effect of changing the light directions from the right and left of the drone and face. The third group of controlled light source tests was conducted using a controlled light source with a diffuser from the right side of the drone and face. The light intensity was measured at five levels. The five levels of tests were repeated for 3200 K and 5500 K. The distance when the subject’s face was recognized was measured at each test.

The third group of controlled light source tests was conducted using a controlled light source with a diffuser from the right side of the drone and face. The light intensity was measured at five levels. The five levels of tests were repeated for 3200 K and 5500 K. The distance when the subject’s face was recognized and measured at each test is shown in Table 3. The actual and sidelight intensities were measured when the drone’s sensor faced the light source and rotated to 90° for the right side.

The fourth group of controlled light source tests was conducted using a controlled light source without a diffuser from the right side of the drone and face. The light intensity was measured at five levels. The five levels of tests were repeated for 3200 K and 5500 K. The distance when the subject’s face was recognized and measured at each test is shown in Table 4. The actual and sidelight intensities were measured when the drone’s sensor faced the light source and when rotated to 90° for the right side.

The fifth group of controlled light source tests was conducted using a controlled light source with a diffuser from the left side of the drone and the face. The light intensity was measured at five levels. The five levels of tests were repeated for 3200 K and 5500 K. The distance when the subject’s face was recognized and measured at each test is shown in Table 5. The actual and sidelight intensities were measured when the drone’s sensor faced the light source and rotated to 90° for the left side.

The sixth group of controlled light source tests was conducted using a controlled light source without a diffuser from the left side of the drone and face. The light intensity was measured at five levels. The five levels of tests were repeated for 3200 K and 5500 K. The distance when the subject’s face was recognized and measured at each test is shown in Table 6; the actual and the side light intensities were measured when the drone’s sensor faced the light source and when rotated to 90° for the left side.

Finally, a set of two experiments was conducted to measure the effect of changing the light directions from the front, right, and left of the drone and face at a fixed distance. The light intensity was measured from three directions. The tests were repeated for 3200 K and 5500 K with and without a diffuser. The distance was fixed to 130 cm when the subject’s face was recognized as shown in Table 7 and Table 8. The actual and sidelight intensities were measured when the light sensors faced the light and when the drone’s sensor rotated to 90° for right and left. The actual and sidelight intensities were measured when the light sensors faced the light and when the drone’s sensor rotated to 90° for right and left.

## 4. Discussion

**Figure 14:**
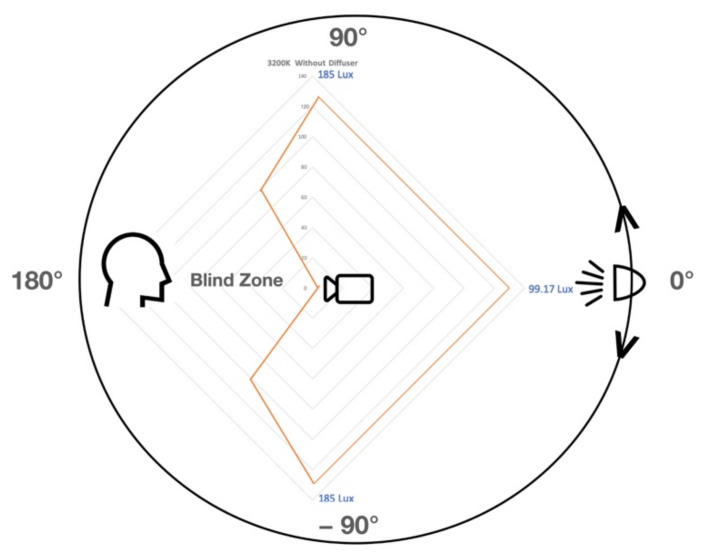
Compare the required light intensity (Lux) for facial recognition of 3200 K light source without diffuser from front, right, and left side for a fixed distance of detection of 130 cm.

The last two sets of experiments were conducted to measure and compare the effects of changing the light directions from the front, right, and left of the drone and face with a fixed distance. The light intensity was measured from three directions. The tests were repeated for 3200 K and 5500 K with and without a diffuser. The distance was fixed to 130 cm when the subject’s face was recognized, as shown in Table 7 and Table 8. The results clearly showed the impact of changing light direction from the two sides to the front side. The light intensity required to recognize the face identity at 130 cm was almost equal when the light was from the left and right with/without a diffuser. The 3200 K light tests that were conducted without a diffuser showed a huge difference of more than 85% of the light intensity required for the same distance when the light direction changed from the front side to right and left. The light intensity required from the front was 99.17 lux, while from the two sides was 185 lux, as shown in Figure 14. For the 3200 K without a diffuser, the light intensity required from the front was 95 lux while from the two sides was 154.17 lux which is around 62.3%. Then, the 3200 K light tests conducted with a diffuser showed a difference of more than 31.4% of the light intensity required for the same distance when the light direction changed from the front side to the right and left sides. The light intensity required from the front was 114.17 lux, while from the two sides was 150. For the 3200 K with diffuser, the light intensity required from the front was 97.5 lux while from the two sides was 133.33 lux, which is around 36.7%. From the above results, it shows clearly that for light without a diffuser, the differences are huge between the front side and two sides, while the light source with a diffuser has a smaller difference compared to without light without a diffuser.

Since a significant correlation was found between facial recognition’s detection distance, light source direction, light intensity, and light color (*p* < 0.05), as shown in Table 9, optimal efficiency of facial recognition for medication delivery could be achieved using the IoT-GLS where it can help the operator or autonomous system to select the best angle of landing based on the light direction and intensity.

**Figure 15:**
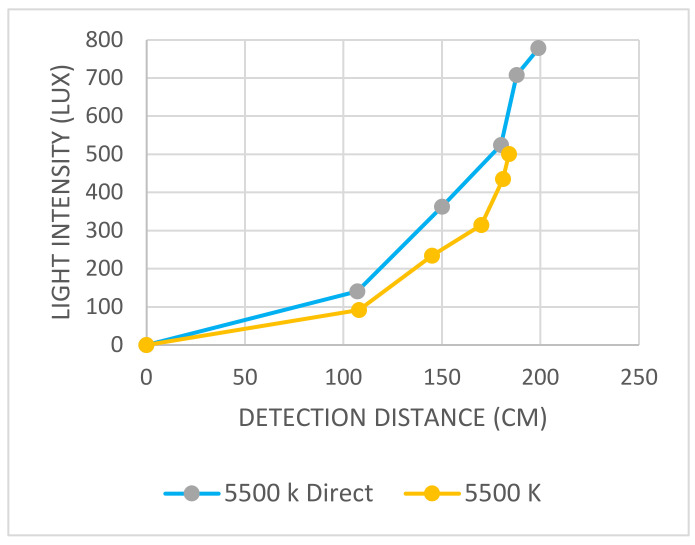
Graph shows the relation between light intensity (Lux) and detection distance (cm) for 5500 K soft light and 5500 K direct light without diffuser.

**Figure 16:**
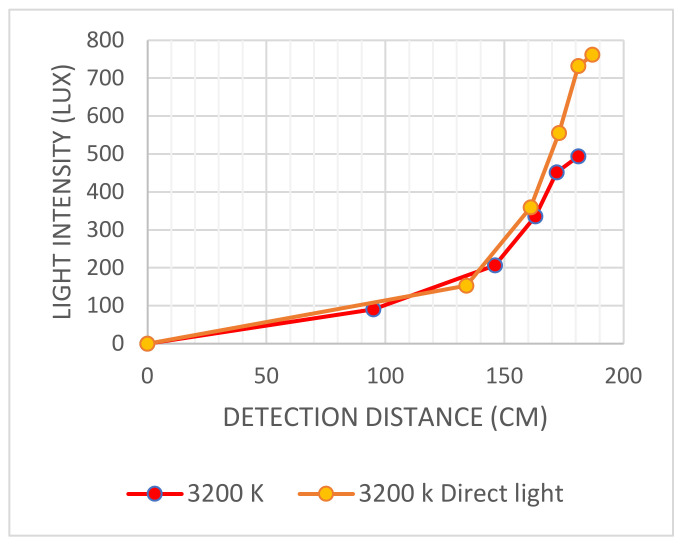
Graph shows the relation between light intensity (Lux) and detection distance (cm) for 3200 K soft light and 3200 K direct light without diffuser.

The highest correlation was found at 5500 K, where the soft light had a longer detection distance, as shown in Figure 15. For the 3200 K direct light, the detection distances were almost the same, as shown in Figure 16.

The main challenge is to use a very lightweight facial recognition camera without Internet connectivity due to the drone’s payload. The Huskylens camera has an on-chip facial recognition feature that fits the requirements and shows a significant accuracy in identifying the patient that exceeds 98% in our experiments in perfect environmental conditions. The facial recognition efficiency was improved by our proposed GLS system. The controllable laser light helps the patient in finding the accurate location in front of the camera.

In our design, there are four limitations. The first limitation is during the day when the sun has an angle of less than 45 degrees during sunrise and from afternoon to sunset. The second limitation is during the night in dark areas with a single LED light source where the light intensity is between 20 and 50 Lux. The third limitation is in a dark environment without any source of light or an insufficient light source where the light intensity is less than 20 Lux. Finally, the fourth limitation is when the user wears a face mask where our camera cannot detect faces.

The proposed solutions to overcome the first and second limitations were to introduce GLS, which provides the best landing angel. For the third limitation, a mini-LED light source can be installed and attached to the drone to flash the light when it detects a dark environment. For the fourth limitation, a Voice Guiding System (VCGU) was added to the system, which, when activated, will request the users to stand in front of the camera and remove their masks. In case of a further failure of detection, we propose to add a contactless NFC card reader RC-522 to the drone, as shown in Figure 5, as well as a keypad. For a more secure experience, the drone’s camera will send a photo of an unauthorized person to the control center and send a video of the delivery process.

Most of the published studies to develop and improve the facial recognition algorithms are focused on developing software to overcome the illumination issues. Our proposed system is developed to be assistive to facial recognition algorithms, especially in an outdoor environment where it is difficult to control environmental conditions. The majority of the published research discusses indoor controlled environments.

## 5. Conclusions

A contactless medical delivery drone was developed and tested successfully to deliver medicine to the patient in a safe mode environment in response to the COVID-19 pandemic. A facial recognition camera was used to detect users’ identity. To overcome the current shortcomings of the medical delivery drone during sunrise, from afternoon to sunset and during night in dark areas where the light intensity is between 20 and 50 Lux, an IoT-GLS enhancement was introduced. The developed IoT mobile app is able to show the percentage of light intensity from four directions and light intensity value from the backside of the camera lens which indicates the light intensity that falls on the patient’s face. Furthermore, results from the experiments found that there is significant correlation between the light direction and intensity with detection distance (*p* < 0.05). The finding enabled the developed IoT-GLS to assist the autonomous system or drone’s operator to select the best angle of landing which consequently increased the distance of face detection to more than two meters. Future work will focus on the effect of a wide range of light color temperatures and improve the processes of medicine delivery, as well as test the GLS on different facial recognition algorithms.

## Figures and Tables

**Table 1:** Compare the relation among light intensity (Lux), light color (Kelvin), and detection distance (cm) for light source with a diffuser.

3200 K		5500 K	
Distance * (cm)	Light Intensity ^ (Lux)	Distance * (cm)	Light Intensity ^ (Lux)
**95**	90.83	108	91.67
**146**	206.67	145	234.17
**163**	335.83	170	314.17
**172**	451.67	181	435
**181**	494.17	184	500.83

* Distance when the subject’s face was recognized. ^ Light intensity measured using the sensors on the medical delivery drone.

**Table 2:** Compare the relation among light intensity (Lux), light color (Kelvin), and detection distance (cm) for light source without diffuser.

3200 K		5500 K	
Distance * (cm)	Light Intensity ^ (Lux)	Distance * (cm)	Light Intensity ^ (Lux)
**134**	152.5	107	140.83
**161**	359.17	150	362.5
**173**	555	180	524.17
**181**	731.67	188	707.5
**187**	761.67	199	778.33

* Distance when the subject’s face was recognized. ^ Light intensity measured using the sensors on the medical delivery drone.

**Table 3:** Compare the relation among light intensity (Lux), light color (Kelvin), and detection distance (cm) for light source with diffuser from right side.

3200 K			5500 K		
Light Intensity ^ (Lux)	Light Intensity ^ Right (Lux)	Distance * (cm)	Light Intensity ^ (Lux)	Light Intensity ^ Right (Lux)	Distance * (cm)
60.83	10	69	56.67	9.17	76
129.17	22.5	120	113.33	18.33	97
193.33	36.67	140	183.33	27.5	123
241.67	46.67	151	231.67	37.5	144
307.5	53.33	183	315	50	174

* Distance when the subject’s face was recognized. ^ Light intensity measured using the sensors on the medical delivery drone.

**Table 4:** Compare the relation among light intensity (Lux), light color (Kelvin), and detection distance (cm) for light source without diffuser from right side.

3200 K			5500 K		
Light Intensity ^ (Lux)	Light Intensity ^ Right (Lux)	Distance * (cm)	Light Intensity ^ (Lux)	Light Intensity ^ Right (Lux)	Distance * (cm)
98.33	10.83	87	91.67	10.83	90
208.33	21.67	127	210	25	128
335.83	31.67	144	339.17	40	153
415.67	41.67	156	462.5	54.17	159
495.83	58.33	164	503.33	59.17	166

* Distance when the subject’s face was recognized. ^ Light intensity measured using the sensors on the medical delivery drone.

**Table 5:** Compare the relation among light intensity (Lux), light color (Kelvin), and detection distance (cm) for light source with diffuser from left side.

3200 K			5500 K		
Light Intensity ^ (Lux)	Light Intensity ^ Left (Lux)	Distance * (cm)	Light Intensity ^ (Lux)	Light Intensity ^ Left (Lux)	Distance * (cm)
60.83	10.83	70	56.67	9.17	75
129.17	24.17	121	113.33	19.17	99
193.33	35.83	141	183.33	34.17	131
241.67	49.17	156	231.67	42.5	147
307.5	51.67	172	315	53.33	177

* Distance when the subject’s face was recognized. ^ Light intensity measured using the sensors on the medical delivery drone.

**Table 6:** Compare the relation among light intensity (Lux), light color (Kelvin), and detection distance (cm) for light source without diffuser from left side.

3200 K			5500 K		
Light Intensity ^ (Lux)	Light Intensity ^ Left (Lux)	Distance * (cm)	Light Intensity ^ (Lux)	Light Intensity ^ Left (Lux)	Distance * (cm)
98.33	12.5	90	91.67	11.67	92
208.33	25	137	210	24.17	124
335.83	35.83	152	339.17	41.67	150
415.67	47.5	158	462.5	51.67	153
495.83	66.67	180	503.33	57.5	162

* Distance when the subject’s face was recognized. ^ Light intensity measured using the sensors on the medical delivery drone.

**Table 7:** Comparison results between the light intensity (Lux) and light color (Kelvin) for a fixed distance of detection of 130 cm for light source without diffuser from front, right, and left side.

3200 K				5500 K		
Direction	Light Intensity ^ (Lux)	Light Intensity ^ Sides (Lux)	Distance * (cm)	Light Intensity ^ (Lux)	Light Intensity ^ Sides (Lux)	Distance * (cm)
**Front**	99.17	99.17	130	95	95	130
**Right**	185	25.83	130	154.17	20.83	130
**Left**	185	25	130	154.17	20	130

* Distance when the subject’s face was recognized. ^ Light intensity measured using the sensors on the medical delivery drone.

**Table 8:** Comparison results between the light intensity (Lux) and light color (Kelvin) for a fixed distance of detection of 130 cm for light source with diffuser from front, right, and left side.

3200 K				5500 K		
Direction	Light Intensity ^ (Lux)	Light Intensity ^ Sides (Lux)	Distance * (cm)	Light Intensity ^ (Lux)	Light Intensity ^ Sides (Lux)	Distance * (cm)
**Front**	114.17	114.17	130	97.5	95	130
**Right**	150	26.67	130	133.33	26.67	130
**Left**	150	27.5	130	133.33	26.67	130

* Distance when the subject’s face was recognized. ^ Light intensity measured using the sensors on the medical delivery drone.

**Table 9:** Results of Pearson correlation coefficient between the 3200 K and 5500 K with and without diffuser.

	3200 K		5500 K	
	With Diffuser	Without Diffuser	With Diffuser	Without Diffuser
**r**	0.9415	0.9782	0.9625	0.9749
**p**	0.016835	0.003851	0.008668	0.004756

## Data Availability

Not applicable.
